# Understanding older patients’ willingness to have medications deprescribed in primary care: a protocol for a cross-sectional survey study in nine European countries

**DOI:** 10.1186/s12877-022-03562-x

**Published:** 2022-11-30

**Authors:** Renata Vidonscky Lüthold, Katharina Tabea Jungo, Kristie Rebecca Weir, Anne-Kathrin Geier, Beatrice Scholtes, Donata Kurpas, Dorothea M. G. Wild, Ferdinando Petrazzuoli, Hans Thulesius, Heidrun Lingner, Radost Assenova, Rosalinde K. E. Poortvliet, Vanja Lazic, Zsofia Rozsnyai, Sven Streit

**Affiliations:** 1 Institute of Primary Health Care (BIHAM), University of Bern, Mittelstrasse 43, 3012 Bern, Switzerland; 2 Graduate School for Health Sciences, University of Bern, Bern, Switzerland; 3 Sydney School of Public Health, Faculty of Medicine and Health, The University of Sydney, Sydney, Australia; 4 Department of General Practice, Faculty of Medicine, Leipzig University, Leipzig, Germany; 5 Research Unit of Primary Care and Health, Department of General Medicine, Faculty of Medicine, University of Liège, Liège, Belgium; 6 Family Medicine Department, Wrocław Medical University, Wrocław, Poland; 7 Institute of Family Medicine and General Practice, University Hospital Bonn, Bonn University, Bonn, Germany; 8 Sezione SNaMID Caserta, Caserta, Italy; 9 Center for Primary Health Care Research, Department of Clinical Sciences, Lund University, Malmö, Sweden; 10 Department of Medicine and Optometry, Faculty of Health and Life Sciences, Linnaeus University, Kalmar, Sweden; 11 Department of Clinical Sciences, Lund University, Malmö, Sweden; 12 Hannover Medical School, Center for Public Health and Healthcare, Hannover, Germany; 13 Department of Urology and General Practice, Faculty of Medicine, Medical University of Plovdiv, Plovdiv, Bulgaria; 14 University Network for the Care sector Zuid-Holland, Leiden University Medical Center, Leiden, The Netherlands; 15 Department of Public Health and Primary Care, Leiden University Medical Center, Leiden, The Netherlands; 16 Health center Zagreb – Centar, Zagreb, Croatia

**Keywords:** Deprescribing, Polypharmacy, Primary care, Survey study, Older adults

## Abstract

**Introduction:**

To reduce inappropriate polypharmacy, deprescribing should be part of patients’ regular care. Yet deprescribing is difficult to implement, as shown in several studies. Understanding patients’ attitudes towards deprescribing at the individual and country level may reveal effective ways to involve older adults in decisions about medications and help to implement deprescribing in primary care settings. In this study we aim to investigate older adults’ perceptions and views on deprescribing in different European countries. Specific objectives are to investigate the patients’ willingness to have medications deprescribed by medication type and to have herbal or dietary supplements reduced or stopped, the role of the Patient Typology (on medication perspectives), and the impact of the patient-GP relationship in these decisions.

**Methods and analysis:**

This cross-sectional survey study has two parts: Part A and Part B. Data collection for Part A will take place in nine countries, in which per country 10 GPs will recruit 10 older patients (≥65 years old) each (*n* = 900). Part B will be conducted in Switzerland only, in which an additional 35 GPs will recruit five patients each and respond to a questionnaire themselves, with questions about the patients’ medications, their willingness to deprescribe those, and their patient-provider relationship. For both Part A and part B, a questionnaire will be used to assess the willingness of older patients with polypharmacy to have medications deprescribed and other relevant information. For Part B, this same questionnaire will have additional questions on the use of herbal and dietary supplements.

**Discussion:**

The international study design will allow comparisons of patient perspectives on deprescribing from different countries. We will collect information about willingness to have medications deprescribed by medication type and regarding herbal and dietary supplements, which adds important information to the literature on patients’ preferences. In addition, GPs in Switzerland will also be surveyed, allowing us to compare GPs’ and patients’ views and preferences on stopping or reducing specific medications. Our findings will help to understand patients’ attitudes towards deprescribing, contributing to improvements in the design and implementation of deprescribing interventions that are better tailored to patients’ preferences.

## Introduction

The high rate of polypharmacy, commonly defined as the regular use of ≥5 medications [1], is a worldwide public health problem. Recent studies have found that the prevalence of polypharmacy in older adults is rising in the last years, ranging from 26 to 40% in Europe [2–5]. There is also evidence that patients with polypharmacy are at higher risk of inappropriate medication use [6]. Inappropriate medication use has been associated with adverse outcomes, including the increased risk for falls [7], adverse drug reactions [8], declined functional ability, cognitive capacity, and nutritional status [9, 10], poor treatment adherence [11], and impaired quality of life [12]. In Switzerland, 21% of patients with polypharmacy take at least one potentially inappropriate medication (PIM) [13]. Indeed, the prevalence of PIMs is high among older adults worldwide [14, 15]. A medication is considered inappropriate when potential harms outweigh potential benefits in an individual [16]. Adverse effects of inappropriate medication mostly affect older adults due to pharmacokinetic and pharmacodynamic changes with age, increasing vulnerability and probability of drug side effects [17, 18]. The increased awareness of the harms associated with polypharmacy has led to research that focuses on deprescribing, which is defined as “the process of withdrawal (or reduction) of an inappropriate medication, supervised by a health care professional with the goal of managing polypharmacy and improving outcomes” (definition adapted from [19]).

Deprescribing should be implemented in primary care routinely for any patient who is affected by inappropriate medication use, especially older adults [20–23]. While the evidence for deprescribing is growing, individual patients face barriers and concerns when it comes to making deprescribing decisions [24]. Previous research has shown patients’ lack of knowledge about the harms of inappropriate polypharmacy is an important barrier, while a good patient-GP relationship acts as an enabler to deprescribing [25, 26]. Additionally, some patients may fear that the offer of deprescribing is an indication that their doctor is withdrawing care or neglecting them [27]. However, the barriers and enablers faced by older adults are highly individual. As shown in Table 1, the Patient Typology was developed by Weir et al. which identified three types of older adults who vary in their attitudes towards medications, preferences for involvement in decision-making, and openness to deprescribing [28]. This can help to understand more deeply how older patients are experiencing their medications and may help to achieve patient-centred decisions about deprescribing.

**Table 1:** Three ‘types’ of older adults from the Patient Typology^1^

*Type 1:*	Positive attitudes towards medicines, left decisions to their doctor or were strongly guided by them, resistant to deprescribing.
*Type 2:*	Ambivalent attitudes towards medicines, preferred a proactive role in decision-making, were open to deprescribing.
*Type 3:*	Gave medicines little thought, deferred decisions to their doctor or companion, unaware deprescribing is an option.

^1^Previous qualitative study developing the Patient Typology [28]

In recent years there has been focus on patients’ hypothetical willingness to have their medications deprescribed. A recent systematic review and meta-analysis found that most of adults (84%) are willing to have a medication deprescribed [29] and similar findings have been shown in Switzerland [25, 30]. Of note, the studies conducted varied in terms of study design, population, and setting. Associations between willingness to deprescribe, clinical and participant characteristics were inconsistent across studies [29]. Furthermore, the literature mostly focuses on individual survey studies rather than systematic studies looking at deprescribing in different countries. Despite the high hypothetical willingness to have medications deprescribed, the literature shows that there is a much smaller percentage of patients, who agree with the statement: “*I feel that I may be taking one or more medications that I no longer need*”. Furthermore, patients also report a high level of satisfaction with their medications [29] and often indicate not being fully aware of the reasons for taking them or the potential harms caused by medications [31]. Despite the growing research on patients’ willingness to deprescribe, it remains unknown which medications patients would like to stop taking and why. Knowing this, will help designing and implementing deprescribing interventions.

Shared decision-making and patient-physician trust play an essential role in taking and implementing deprescribing decisions [32, 33]. Little is known about patients and health professionals deprescribing preferences and how these preferences compare. A recent study [33] found that patients seem to prefer continuing the use of sedatives and pain killers, but prescribers would rather discontinue these. However, this study was restricted to patients with cognitive disorders, younger than 60 years of age. In this context, it is important to better understand how GPs’ deprescribing suggestions are aligned with their patients’ preferences and how the patient–GP relationship influences deprescribing decisions. Having a better understanding of this will help to reduce disagreement in clinical practice by developing interventions that consider eventual differences [34].

While most of the literature on deprescribing focuses on prescription drugs only, for optimal medication management, GPs should be aware of all the medications used, including such supplements. Herbal and dietary supplements can be PIMs and are commonly used in many countries, including Switzerland [3, 35–39]. For instance, multivitamins are among the most frequently used PIMs [22, 40]. According to the Beers list and STOPPFrail criteria, Ferrous sulfate (iron), multivitamins, and caffeine are examples of PIMs and should be discontinued when prescribed for prophylaxis rather than treatment [41, 42]. Patients are commonly unaware of the potential risks of self-medication [36] and the use of such supplements is often not disclosed to GPs [39]. In this study we focus on supplements (e.g., multivitamins, vitamin D, calcium, iron, magnesium) as they are commonly used over a longer period, as compared to other medications (e.g., cold and flu medications) that can be bought over the counter in Switzerland.

### Study objectives

The overall aim of this study is to investigate older adults’ perceptions and views on the use and deprescribing of prescription medications and supplements in different European countries.

Specific objectives for all participating countries are:

1. To explore older patients’ views on deprescribing and compare how they differ by country.
2. To assess patients’ willingness to have medications deprescribed by medication type.
3. To analyse if and how patients’ hypothetical deprescribing decisions are associated with the three types of the Patient Typology (a qualitative framework).
4. To analyse the association between patients’ perceived trust and relationships with their GP and their willingness to make deprescribing decisions.

Additional objectives for Switzerland, where we do a patient-GP matched survey and collect additional data on herbal and dietary supplements, are:

1. To compare patients’ and GPs’ hypothetical deprescribing decisions and to examine the role of patient-provider relationships with regards to the agreement between patients and GPs.
2. To explore the views of patients on the use and on the reduction or stopping of herbal and dietary supplements.

## Methods and analysis

### Study design

**Fig. 1:**
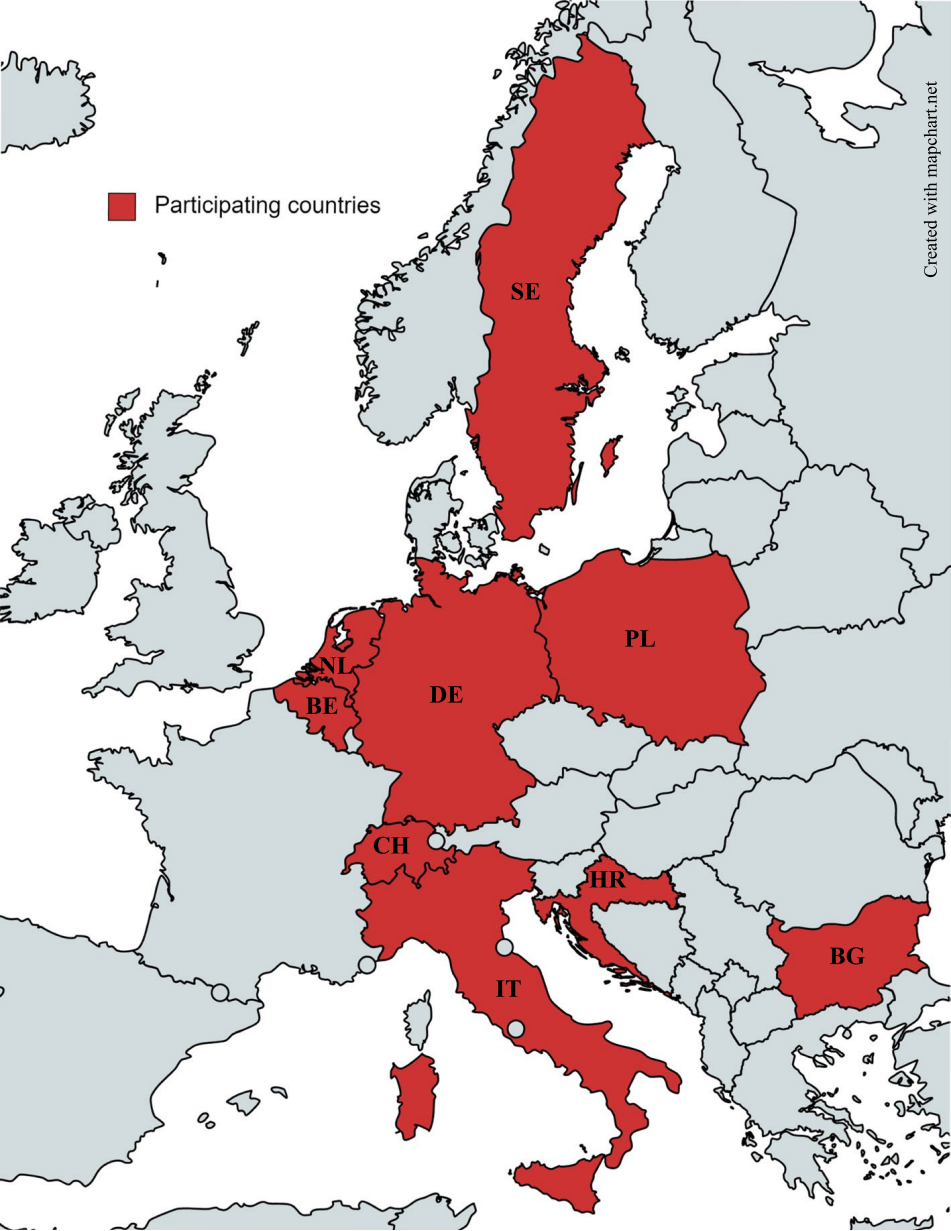
Map of participating countries created with MapChart.net. Maps created with MapChart can be freely used, edited and modified for publications, as long as mapchart.net is referenced (https://mapchart.net/terms.html, accessed July 15, 2022)

This cross-sectional study contains two parts: Part A and Part B. Part A involves nine European countries (Fig. 1) with anonymous data collection on older adults’ willingness to have medications deprescribed. Part B is a nested sub-study in Switzerland only, which extends Part A by collecting additional data from older patients and GPs.

In both Part A and Part B, we are using a questionnaire to assess patients’ willingness to have their medications deprescribed, Patient Typology, and other relevant sociodemographic and clinical information on older patients with polypharmacy. For Part B, an additional questionnaire will be distributed to GPs in Switzerland, which will contain questions about the patients’ medications and the GP-patient relationship. Patients in Part B will be asked about their use of herbal and dietary supplements and their willingness to stop or reduce them. Table 2 shows further details.

**Table 2:** Summary of Part A and B of the project

	Part A	Part B
Countries involved	Switzerland, Germany, Poland, Sweden, French-speaking part of Belgium, Bulgaria, Italy, Croatia, and Netherlands	German-speaking part of Switzerland
Anonymization	Anonymized	Pseudonymized
Subjects	Patients	Patients and their GPs
Questionnaires used	Patient questionnaires in local language(s)	Patient questionnaire including part on herbal and dietary supplements, GP questionnaires
Number of recruited GPs	10 per country	35 in the Swiss German part of Switzerland
Role of the GPs	Screen and recruit eligible patients	Screen and recruit eligible patients, complete questionnaires themselves
Number of recruited patients	100 per country (10 per GP) = 900 in total	5 per GP = 175 in total

### Setting

The study will be conducted in primary care settings in nine European countries. It is coordinated by the central study team in Switzerland at the Institute of Primary Health Care (BIHAM) of the University of Bern and conducted in collaboration with a group of GPs from the European General Practice Research Network (EGPRN) – a proven successful collaboration [43–45]. Seventeen National Coordinators from 13 different countries were invited to participate in the study, of which 11, coming from 8 different countries, accepted to participate (Fig. 1). Four National Coordinators are participating from different locations in Germany. The list of participant countries is subject to changes. 

### Participants

#### Eligibility criteria

For both parts, patients will be included if they are 65 years or older and have polypharmacy (taking ≥5 prescribed medications regularly). Patients are not eligible if they are unable to give informed consent or if they do not reside in one of the participating countries. For Part B, due to language reasons, the inclusion criterium for the GPs participating in the additional data collection is to be a practicing GP in the German-speaking part of Switzerland.

#### Screening and recruitment

Starting in May 2022, through the networks of the National Coordinators at each site, we aim to recruit GPs who will in turn recruit patients. For study Part A, our goal is to recruit a total of 900 primary care patients, which corresponds to approximately 100 patients per country (around 10 per GP). For Part B we will recruit an additional 35 GPs, who will invite five patients each to respond to a questionnaire and will also complete a questionnaire themselves for each of the recruited patients.

For Part A and B, primary care patients will be recruited through their GP. GPs will be recruited through the National Coordinators at the participating sites and the study team at BIHAM in Switzerland. GPs will be given screening criteria to be able to screen and recruit primary care patients in their practice. Screening criteria will be sent to all the participating GPs. Screening and recruitment of the patients will take place during the regular consultation hours of the GPs. They will be instructed to screen their patients consecutively (e.g., on a work half-day) to reduce selection bias. In the Netherlands, GPs are able to screen their patients in their electronic medical records and then invite a random sample of them.

Due to the anonymous design of Part A, patients will give their informed consent by replying to the question “by clicking yes here, I agree to participate in this study”. If they click “no”, they cannot participate in the study. For Part B, patients will have to give their written informed consent to participate. As soon as all questionnaires from one GP practice have been completed, the GP will return the questionnaire to the study team in Switzerland or to the respective National Coordinator of the participating sites.

### Questionnaire

Cross-cultural adaptation of the questionnaire will be carried out by the National Coordinator in each participating country. Translations will be validated by performing back-translations to English and solving eventual inconsistences.

For patients (Part A and B), the questionnaire contains questions on demographic characteristics, educational level, housing and living situation, health literacy, medication management and information on life circumstances. Patients will be asked to specify any medications they would potentially discontinue, for what reason, and the support they would need to do this. Furthermore, the survey will contain questions on trust in the physician and questions on the Patient Typology. In Part B, patients will also be asked about herbal and dietary supplements. In Part B, GPs will be asked to attach the patient’s medication list to the questionnaire and indicate which medications they would be willing to deprescribe. The GP questionnaire will contain sociodemographic questions, questions about work practices, and decision-making preferences (“GP profile”) based on previous qualitative research [46]. Details on the individual components of the questionnaire, and how they related to the study objectives, are provided in Table 3.

**Table 3:** Study objectives and survey tools

Objective	Data collection tool
**Part A: European data collection**	
1) To explore patients’ views on deprescribing specific medications and compare how they differ by country.	1) Two questions from the revised Patients’ Attitudes Towards Deprescribing (rPATD) questionnaire [47].
2) To assess patients’ willingness to have medications deprescribed by medication type.	2) Questions on hypothetical deprescribing decisions related to patients’ own medications.
3) To analyse if and how patients’ hypothetical deprescribing decisions are associated with the three types of the Patient Typology (a qualitative framework).	3) Questions based on the typology of three ‘types’ of older adults (the Patient Typology) [28].
4) To analyse the association between patients’ perceived trust and relationships with their GP and their willingness to make deprescribing decisions.	4) Questions from the abbreviated Wake Forest Trust in Physician Scale [48].
**Part B: Patient-GP data collection in Switzerland**	
5) To compare patients’ and GPs’ hypothetical deprescribing decisions and to examine the role of patient-provider relationships with regards to the agreement between patients and GPs.	5) GP questionnaire asking if and why GPs would stop/reduce any of their patients’ medications, questions regarding their relationship with the patient, and sociodemographic questions. We also use adapted questions from the Control Preference Scale [49], GP typology [46], and Prescribers’ Perceptions of Medication Discontinuation Survey [50].
6) To explore the views of older adults on the use and deprescribing of herbal and dietary supplements.	6) Questions on the use of herbal or dietary supplement by patients.

### Data collection and data management

Paper and online versions of the questionnaire will be available to participants. Part A is anonymized and thus complies with the European General Data Protection Regulation (GDPR). Part B is pseudonymized and not anonymized, as we need to be able to match GPs and patients for the analysis.

We are programming the online survey using REDCap [51], which provides role-based user access control and audit trails [52]. The questionnaire will either be entered into REDCap by the National Coordinators, or the participant can fill in the survey directly online using REDCAp survey function. Only selected members of the research team will have access to the full database in REDCap.

### Sample size

A recent systematic review found that 84% of patients strongly agree to have one or more of their medications deprescribed [29]. In Switzerland the results were similar with 77% of patients agreeing to deprescribe one or more of their medications [25]. For the sample size calculations, we used the more conservative estimate of 77%. The sample size calculation accounts for the clustered nature of data for patients within the same GP (ICC = 0.10), which is more conservative than the Intra-cluster correlations (ICC) of 0.01 to 0.05 that were reported for binary outcomes in cluster clinical trials of older individuals [53]. We did all sample size calculations using the power one proportion function in Stata, which allows to account for the clustered nature of the data.

#### **Calculations for Part A**

Based on the assumption that 77% of patients would be willing to deprescribe (yes/no), assuming an ICC of 0.10, we need a total of 80 clusters (i.e., GPs recruiting patients and distributing surveys, around 8 per site), and 8 patients recruited per GP, to have an effect size of 0.06 at a power of 0.90. To account for potential missing data, we increased the number of GPs per site to 10 and the number of patients per cluster to 10.

#### **Calculations for Part B**

This part of the study is powered for the GP-patient agreement related to deprescribing specific medications. In line with the literature on the agreement between GPs and patients with regards to which medication to (dis-)continue, in around half of the cases patients and GPs were in agreement regarding which medications to continue [33]. Assuming an ICC of 0.10, we need a total of 33 clusters (GPs) and 4 patients with a minimum of 5 medications each per cluster to have an effect size of 0.10 at a power of 0.90. Overall, to account for missing data, we aim to recruit 35 GPs from the German-speaking part of Switzerland, who will be instructed to recruit 5 patients each. This will result in around 175 patients and a sufficient number of medications that were rated by both GPs and patients (willing to deprescribe yes/no). There will be a minimum of 875 medications if each study participant has a minimum of 5 medications. Likely, there will be more medications to compare though, since in a previous study with a similar study population the mean number of medications was 8 [25].

### Statistical analysis

#### Part a

From the rPATD [47], we are using the question *‘If my doctor said it was possible, I would be willing to stop one or more of my regular medications’* to assess the primary outcome for objective 1. In a sensitivity analysis, we also use the question ‘*I would like to try stopping one of my medicines to see how I feel without it*’ from the rPATD. The rPATD questions with 5-point Likert scale responses will be dichotomized into *“strongly agree/agree”* versus *“unsure/disagree/strongly disagree”*. If patients agree or strongly agree with this statement, they will be considered to be willing to deprescribe. Descriptive statistics will report baseline characteristics of the sample stratified by willingness to deprescribe. Where appropriate, the t-test and Chi-square test will be used to compare participants who were willing to deprescribe versus not willing to deprescribe. To explore the patients’ willingness to have medications deprescribed, we will assess univariate and multivariate associations between sociodemographic and clinical characteristics (e.g., age, sex, medication management, living status, education level, number of medications, etc.) and their willingness to have medications deprescribed using mixed-effects logistic regression models. Models will be adjusted for clustering effects at GP and country level. We will use a hypothesis-driven approach to select the confounders we have to adjust for. To analyse how the views on deprescribing differ among the participating sites, we will use the same regression model, but stratify by country.

For objective 2, we will descriptively analyse which types of medications patients were most likely to report as willing to stop or reduce from their own medication use. We will also compare the reasons provided for stopping or reducing by medication type. Using multivariate mixed-effects logistic regression analyses, we will also investigate patients’ sociodemographic and clinical characteristics associated with being willing to have certain medication types deprescribed.

For objective 3, we will assess the association between the three “types” of the Patient Typology *Dimension* participants identify with and patients’ hypothetical deprescribing decisions. To do so we will use a multivariate mixed-effects logistic regression model that will be adjusted for patient sociodemographic and clinical characteristics and clustering at the GP and country level.

For objective 4, we will analyze the associations between patient-provider relationships (reported by patients) and patients’ willingness to make deprescribing decisions using multivariate mixed-effects logistic regression analyses that will be adjusted for patient sociodemographic and clinical characteristics and clustering at the GP and the country level.

#### Part B

For objective 5, we will analyse the agreement between patients’ and GPs’ hypothetical deprescribing decisions. We will use descriptive statistics to describe the percentage of (dis) agreement between patients and GPs and which types of medications they most commonly (dis) agree about. Logistic regression models will be used to assess the association between GP-patient trust, patient and GP characteristics, and the agreement between GPs’ and patients’ willingness to make hypothetical deprescribing decisions.

Finally, for objective 6, we will investigate the use, beliefs, and motivations of patients for taking herbal and dietary supplements and their willingness to stop or reduce using such supplements. Descriptive statistics will be used to determine the percentage of patients who use supplements. Logistic regression models will be used to assess the association between patients’ demographic, behavioural, and health characteristics, the use of supplements, and patients’ willingness to deprescribe those. The analyses will be adjusted for clustering at the GP level.

Baseline characteristics will be presented in proportions (categorical variables) and means ± SD (or medians and IQR) (continuous variables). A two-sided *p*-value of 0.05 will be considered statistically significant. Analyses will be performed with STATA 16.1 (StataCorp, College Station, TX, USA).

## Discussion

Overall, the aim of our study from 9 European countries about older primary care patients’ willingness to have medications deprescribed is to better understand patients’ attitudes towards deprescribing at the individual and country level. Eventually, the study’s goal is to inform effective ways to involve older adults in decisions about their pharmacological treatment. To the best of our knowledge, this is one of the first studies comparing patients’ willingness to have medications deprescribed across countries. It will also be one of the first studies to look at both the willingness to have prescription medications and supplements stopped or reduced. A better understanding of the enablers and barriers of the willingness to deprescribe in older patients with polypharmacy by answering the questions raised in this project, may contribute to improvements in the design and implementation of deprescribing interventions that are better tailored to patients’ preferences. This in turn will directly help GPs and other health professionals to optimise the process of approaching and implementing deprescribing in patients with polypharmacy. This will provide a better understanding of the management of polypharmacy and medication optimization, especially in older individuals. Ultimately this may improve patients’ overall health, reduce adverse effects caused by inappropriate polypharmacy, and eventually reduce the burden of polypharmacy on different health care systems in Europe and worldwide.

This study is strengthened by its approach to patient and public involvement, National Coordinators are partners of the Swiss central study team, and they have helped shape several aspects of the study, such as the recruitment strategy. Therefore, we aligned the data collection with all countries, to ensure feasibility of the project in regard to the format of the questionnaire, timeline of the data collection, etc. Each of the National Coordinators signed a Research Collaboration Agreement, in which the duties, tasks, qualifications for co-authorship and data use are clarified. More details on the decisions taken together with the National Coordinators are shown in Table 4. Although in Switzerland, primary care research is gaining attention, it is still a difficult context in which to conduct research. However, our team succeeded in overcoming such difficulties when conducting research involving GPs and patient recruitment in the past [43, 54]. The questionnaires (both paper-based and online version) used in this study have been piloted with 6 patients and 4 GPs and were revised based on their feedback.

**Table 4:** Involvement of National Coordinators in study design and planning of the data collection

Question	Decisions made
Timeline	Data collection begins in May 2022 in Switzerland and in June 2022 in the other countries (depending on how the COVID-19 situation evolves).
Data collection format	Offer patients both online and on paper questionnaires so that they can chose a suitable format.
Survey tool	The questionnaire will be translated into German, French, Italian, Bulgarian, Swedish, Croatian, Polish, and Dutch, and culturally adapted by the National Coordinators.
Data collection procedure	GPs will collect the questionnaires from patients and send them to the National Coordinators, who will enter the data into REDCap.

### Strengths and limitations

As this will be a cross-sectional study design and we will ask hypothetical deprescribing questions, the directionality of the associations cannot be confirmed. Nevertheless, our study will add important information to the literature comparing GPs’ and patients’ preferences on deprescribing specific medication types. We are limited by GDPR and the available funding and therefore cannot compare GPs’ and patients’ preferences in all participating countries but will focus on Switzerland. For Part A, due to the irreversible anonymization, we are not able to track the response rate nor are we able to adjust the analyses for the clustering effect at the GP level. However, we will be able to adjust the analyses for GP-level variables.

This study is strengthened by the fact that it will investigate which specific medications patients would prefer to deprescribe and for which reason. Another strength will be the international study design with 12 participating sites, which will allow us to compare patient perspectives on deprescribing from different European countries.

## Data Availability

The dataset used and analysed during the current study is available from the corresponding author on reasonable request. The questionnaires used in this study are available upon request.
